# Crack-Configuration Analysis of Metal Conductive Track Embedded in Stretchable Elastomer

**DOI:** 10.3390/mi9030130

**Published:** 2018-03-15

**Authors:** Tomoya Koshi, Eiji Iwase

**Affiliations:** Department of Applied Mechanics, Waseda University, 3-4-1 Okubo, Shinjuku-ku, Tokyo 169-8555, Japan; koshi@akane.waseda.jp

**Keywords:** crack configuration, metal conductive track, stretchable elastomer, flexible electronic device, stretchable electronic device

## Abstract

This paper reports the analysis of the crack configuration of a stretched metal conductive track that is embedded in a stretchable elastomer. The factor determining the crack configurations is analyzed by modeling as well as experiments. The modeling analysis indicates that the crack configuration is determined by the ratio of the elongation stiffness of the track and elastomer, and is classified into two types: multiple-crack growth and single-crack growth. When the track stiffness is considerably lower than the elastomer stiffness, multiple-crack growth type occurs; in the opposite case, single-crack growth type occurs. Hence, to verify the modeling analysis, metal conductive tracks with different thicknesses are fabricated, and the cracks are studied with respect to the crack width, number of cracks, and crack propagation speed. In this study, two conventional metal-track shapes are studied: straight-shaped tracks with track thickness of 0.04–1.17 µm, and wave-shaped tracks with track thickness of 2–10 µm. For straight-shaped tracks, multiple-crack growth type occurred, when the track thickness was 0.04 µm, and the crack configuration gradually changed to a single crack, with the increase in the track thickness. For wave-shaped tracks with track thickness of 2–10 µm, only single-crack growth type occurred; however, the crack propagation speed decreased and the maximum stretchability of the track increased, with the increase in the track thickness.

## 1. Introduction

Of late, many research groups have been developing flexible or stretchable electronic devices [1,2,3], such as stretchable displays [4,5], devices fixed to the human skin [6,7,8,9,10,11], and neural interfaces devices that are embedded in animals [12]. As metal conductive tracks are one of the critical components for achieving device flexibility or stretchability, various types of metal tracks, such as straight-shaped metal tracks with microcracks [13,14,15,16], straight-shaped metal tracks on a wavy surface [17,18], and wave-shaped metal tracks [19,20,21,22], have been researched. Straight-shaped tracks with microcracks are stretchable and conductive with randomly distributed tribranched microcracks on the tracks; the track thickness is several tens or hundreds of nanometers, and the metal track layer is directly deposited on a stretchable elastomer substrate by thermal or electron-beam deposition [12]. Straight-shaped metal tracks on a wavy surface are fabricated by direct metal deposition on a prestretched elastomer substrate and can be stretched by the deformation of the wavy surface of the elastomer [17]. Wave-shaped metal tracks can be stretched by the deformation of the wave-shape. The thickness of the metal layer is serval micrometers, and it is fabricated by plating or laminating a metal foil and a stretchable elastomer sheet [19,22].

In previous studies, the observed crack configurations of stretched metal tracks differ considerably. For example, many micro cracks were observed in some studies [12,13,14,15,16]; whereas, few large cracks, which propagated and crossed the metal track perpendicular to the stretching direction, were observed in the others [19,20,21]. However, the factors determining the crack configurations are still not clear. Understanding these factors can contribute to the development and improvement of flexible or stretchable electronic devices because crack configurations affect both the flexibility and stretchability of a metal conductive track. In addition, this understanding can contribute to related studies, such as self-healing methods for a cracked metal track [23,24].

In this study, to analyze the factors that determine the crack configurations, modeling as well as experiments were utilized. In the experiments, we studied two conventional metal-track shapes: straight-shaped metal tracks and wave-shaped metal tracks. For straight-shaped tracks, thinner tracks with thickness of several tens to hundreds of nanometers are generally used to achieve the stretchability using out-of-plane deformation by tribranched microcracks or a wavy surface. For wave-shaped tracks, the in-plane deformation of the wave-shape is mainly used for stretchability; hence, thicker tracks with a thickness of several micrometers are generally used. Therefore, in this study, straight-shaped tracks with track thickness of 0.04–1.17 µm and wave-shaped tracks with track thickness of 2–10 µm were fabricated, and the cracks were studied with respect to the crack width, number of cracks, and crack propagation speed.

## 2. Material and Methods

### 2.1. Modeling Analysis

**Figure 1:**
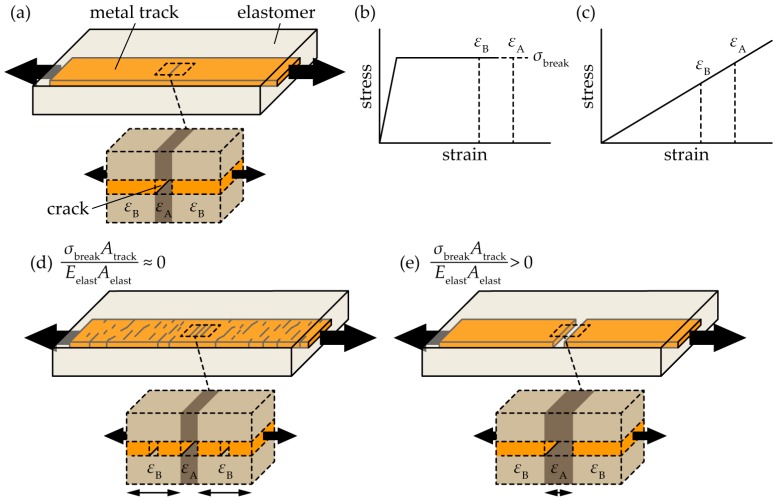
Schematic of the crack-configuration modeling analysis. (**a**) Cracked metal track embedded in a stretchable elastomer; (**b**) simplified stress-strain curve of a metal track layer; (**c**) simplified stress strain-curve of a stretchable elastomer; (**d**) schematic of a multiple-crack growth type; and, (**e**) schematic of a single-crack growth type.

The factors determining the crack configurations were analyzed by modeling. Figure 1a displays the schematic of a cracked metal track embedded in a stretchable elastomer. A small crack is caused in the track, and both the track and the elastomer are deformed by a constant balanced force. In the model, the strain in the cracked region is denoted as *ε*_A_, and that in the non-cracked region as *ε*_B_. We assumed that all of the strains are uniform over each region, and that *ε*_A_ is larger than *ε*_B_, as shown in Figure 1b,c. These figures depict the simplified stress-strain curves of a metal track and stretchable elastomer, respectively. For flexible or stretchable electronic devices, gold or copper are used as conductive tracks, and polydimethylsiloxane (PDMS) or polyurethane (PU) are used as stretchable elastomer layers. Therefore, the stress-strain curves are simplified, based on the material. In the model, the balanced force around the boundary between the cracked and non-cracked regions is represented by

(1) $$\varepsilon_{A}E_{\mathrm{elast}}A_{\mathrm{elast}}=\sigma_{\mathrm{break}}A_{\mathrm{track}}+\varepsilon_{B}E_{\mathrm{elast}}A_{\mathrm{elast}}$$

where *E*_elast_, *A*_elast_, *A*_track_, and *σ*_break_ are the Young’s modulus of the elastomer, cross-sectional area of the elastomer, cross-sectional area of the track, and breaking stress of the track, respectively. Focusing on *ε*_A_ and *ε*_B_, Equation (1) is represented as

(2) $$\varepsilon_{A}-\varepsilon_{B}=\frac{\sigma_{\mathrm{break}}A_{\mathrm{track}}}{E_{\mathrm{elast}}A_{\mathrm{elast}}}$$

The left of Equation (2) is the difference between *ε*_A_ and *ε*_B_, and the right is the ratio of the elongation stiffness of the track and elastomer. Therefore, Equation (2) indicates that the difference between *ε*_A_ and *ε*_B_ is determined by the ratio of the elongation stiffness. In particular, when the ratio of the elongation stiffness is considerably small, then the stiffness of the track is considerably lower than that of the elastomer, i.e.,

(3) $$\varepsilon_{A}-\varepsilon_{B}\approx 0$$

In this case, the values of *ε*_A_ and *ε*_B_ are nearly the same; hence, we consider that other cracks are caused in the non-cracked region, as the track is stretched further, as shown in Figure 1d. In this paper, we refer to this crack configuration as a multiple-crack growth type. On the other hand, when the ratio of the elongation is greater than zero, we consider that a crack already caused in the track increasingly propagates, as the track is stretched further, as shown in Figure 1e. We refer to this crack configuration as a single-crack growth type.

To examine the relationship between the crack configurations and the ratio of the elongation stiffness, the value of the ratio was calculated, with reference to previous studies. In the calculation, we assumed that the widths of the track and elastomer are considerably greater than the thicknesses of the track and elastomer; hence, Equation (2) is represented as

(4) $$\varepsilon_{A}-\varepsilon_{B}=\frac{\sigma_{\mathrm{break}}t_{\mathrm{track}}}{E_{\mathrm{elast}}t_{\mathrm{elast}}}$$

where *t*_track_ and *t*_elast_ are the thicknesses of the track and elastomer, respectively. Table 1 shows the calculated values of the ratio of the elongation stiffness, using Equation (4); the values that are used in Table 1 are as follows: *σ*_break_ of gold was 0.3 GPa [25], *σ*_break_ of copper was 0.2 GPa [26], and *E*_elast_ of PDMS was 1.3 MPa [27]. Table 1 indicates that the multiple-crack growth type is often observed, when the value of the ratio of the elongation stiffness is lower than approximately 0.1. On the other hand, the single-crack growth type is often observed, when the value of the ratio is higher than approximately unity.

### 2.2. Fabrication and Experimental Setup

To verify the modeling analysis, metal conductive tracks with different ratios of the elongation stiffness were fabricated. This ratio was varied by changing the thickness of the metal track. In this study, two conventional metal-track shapes were studied: straight-shaped tracks with track thicknesses of 0.04–1.17 µm, and wave-shaped tracks with track thickness of 2–10 µm. In previous studies on straight-shaped tracks, a metal track layer was deposited on a stretchable elastomer layer by thermal or electron-beam deposition. However, a stretchable elastomer, such as PDMS and PU, changes its mechanical property around the boundary between the track and elastomer, due to thermal damage. In this case, the mechanical property is unclear, and it is difficult to comprehend the factors determining the crack configurations. In both the thermal deposition and transfer methods, some microcracks might be pre-formed in the metal layer; however, the transfer methods is much better because of the no thermal damage. Moreover, if there are the pre-formed microcracks, then the pre-formed microcracks have little effect on the crack configuration. For the multiple-crack growth type, new microcracks are caused in addition to the pre-formed microcracks. On the other hand, for the single-crack growth type, some of the pre-formed microcracks propagate and become the large cracks. Therefore, in this study, a transfer method, in which the metal layer was transferred onto the elastomer, was used. For the wave-shaped metal track, commercially available rolled copper foil was used as the metal track layer. PU was used as the elastomer layer, for both straight-shaped and wave-shaped tracks.

**Figure 2:**
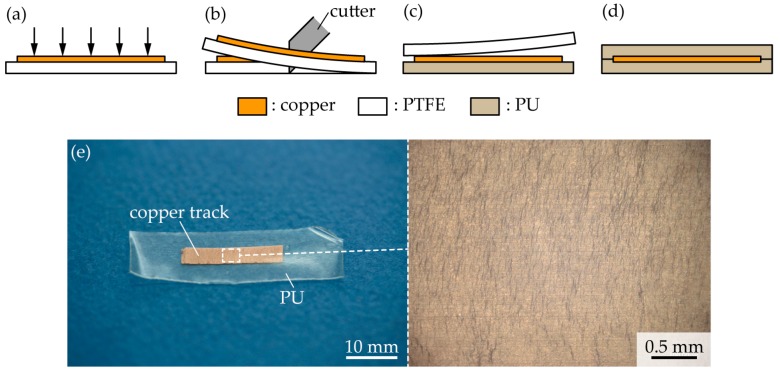
Fabrication of a straight-shaped copper track embedded in PU. (**a**) Thermal deposition of a copper layer on a polytetrafluoroethylene (PTFE) sheet; (**b**) Cutting of the PTFE sheet; (**c**) Transfer of the copper track onto a polyurethane (PU) tape; (**d**) Lamination of another PU tape; and, (**e**) Optical images of the fabricated copper track in PU.

Figure 2a–e depict the fabrication process of a straight-shaped track. Initially, a polytetrafluoroethylene (PTFE) sheet was cut into 20 mm × 30 mm sheets, and a copper layer was deposited on these PTFE sheets by a thermal evaporation system (SVC-700TMSG/7PS80, Sanyu Electron Co., Ltd., Tokyo, Japan), as shown in Figure 2a. The track thickness, *t*_track_, was 0.04 µm, 0.10 µm, 0.18 µm, 0.53 µm, and 1.17 µm, respectively. Further, the PTFE sheet was cut in the shape of a track with a width of 3 mm (Figure 2b), and was pasted onto a PU tape (Tegaderm, 3M, Meipplewood, MN, USA). The thickness of the PU tape was 0.05 mm. The PTFE sheet was then peeled off from the PU tape, and the copper layer was transferred from the PTFE sheet to the PU tape (Figure 2c). Finally, another PU tape was pasted on the copper layer, as shown in Figure 2d, and the PU layer was cut into a sample shape with a width of 10 mm (Figure 2e). The calculated ratios of the elongation stiffness of each sample were 0.03, 0.07, 0.12, 0.35, and 0.78, at *t*_track_ = 0.04 µm, 0.10 µm, 0.18 µm, 0.53 µm, and 1.17 µm, respectively. In the calculation, an *E*_elast_ of 3 MPa was used for the PU tape.

**Figure 3:**
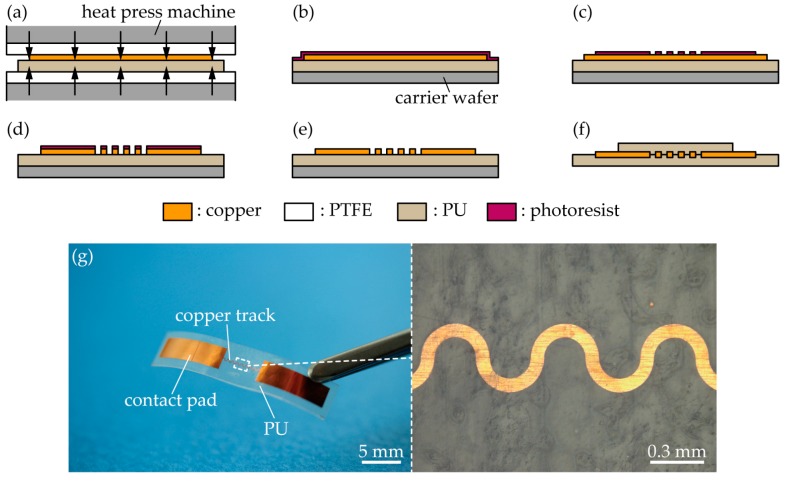
Fabrication of a wave-shaped copper track embedded in PU. (**a**) Lamination of a copper foil on a PU sheet; (**b**) Spin-coating of a photoresist on the copper foil; (**c**) Development and patterning of the photoresist; (**d**) Wet-etching of the copper foil; (**e**) Removal of the photoresist; (**f**) Lamination of a PU tape on the structured copper; and, (**g**) Optical images of a wave-shaped copper track in PU.

Figure 3a–g show the fabrication process of a wave-shaped copper track. Initially, rolled copper foils (The Nilaco Co., Tokyo, Japan) of various thicknesses (*t*_track_ = 2 µm, 4 µm, 6 µm, 8 µm, and 10 µm) were thermally laminated onto a PU sheet (Platilon 4201, Covestro AG, Leverkusen, Germany), as shown in Figure 3a. The thickness of the PU sheet was 0.05 mm. In the lamination process, the copper foil was surface-modified by a plasma cleaner (PDC-32G, Harrick Plasma, New York, NY, USA); the copper foil and PU sheet were heated at 100 °C for 3 min without pressure, and was then pressed at approximately 0.4 MPa, at 170 °C for 3 min. Subsequently, the copper foil was structured by the photolithography process. Photoresist was spin-coated onto the copper foil (Figure 3b), and patterned into a wave-shaped track and contact pads (Figure 3c). Further, the copper layer was wet-etched (Figure 3d) and the photoresist was stripped off (Figure 3e). A PU tape was laminated only on the wave-shaped track, as shown in Figure 3f. Finally, the individual samples were separated. Figure 3e shows the fabricated wave-shaped copper track. The dimensions of each sample were 25 mm by 5 mm. The width of the copper track was 75 µm, and it was arranged between two large contact pads, which were 5 mm apart. The radius of the track was 150 µm. The calculated ratio of the elongation stiffness of each sample was 0.89, 1.78, 2.67, 3.56, and 4.45 at *t*_track_ = 2 µm, 4 µm, 6 µm, 8 µm, and 10 µm, respectively. In the calculation, 3 MPa and 6 MPa were used as the *E*_elast_ values of the PU tape and PU sheet, respectively.

**Figure 4:**
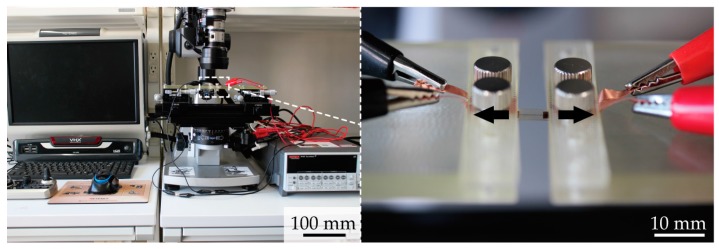
Images of the experimental setup.

Figure 4 displays images of the experimental setup. A sample were mounted onto movable stages. For wave-shaped tracks, the sample was clamped at the contact pads, and electronically connected to a source meter (2614B, Keithley Instruments, Cleveland, OH, USA). The resistance of the wave-shaped track was measured by four probe methods, for detecting crack propagation. The sample was stretched gradually by moving the stages. The crack formation was observed with an optical microscope (VHX-2000, Keyence Corporation, Osaka, Japan).

## 3. Result and Discussion

### 3.1. Straight-Shaped Track 

**Figure 5:**
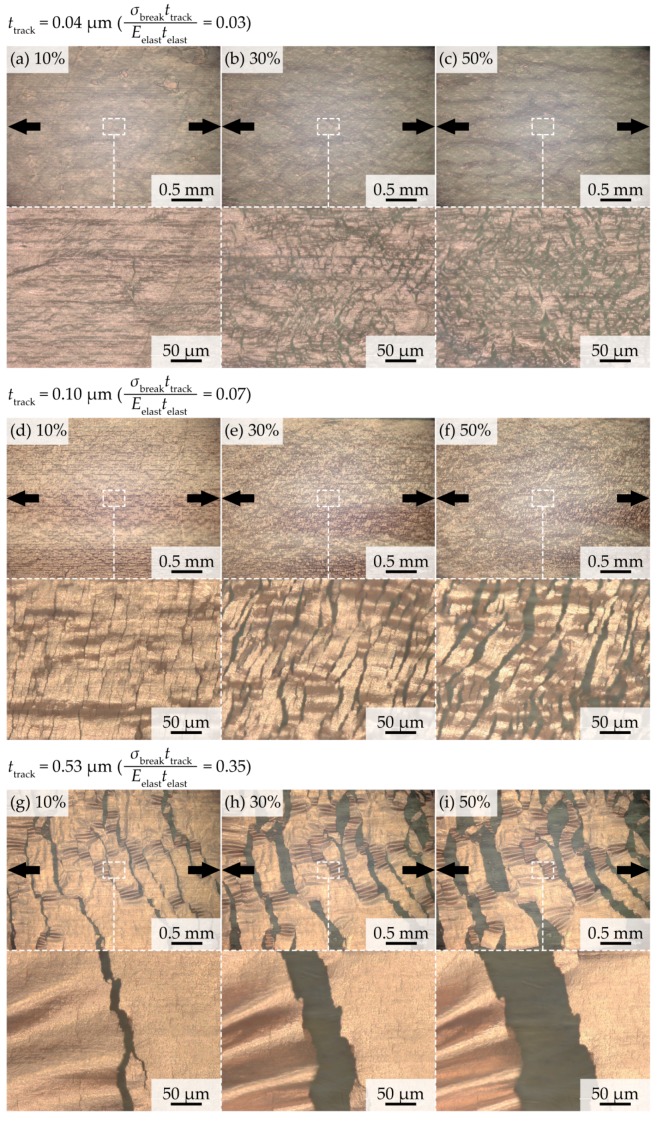
Series of optical images of a cracked copper track in PU with track thicknesses of (**a**–**c**) 0.04 µm; (**d**–**f**) 0.53 µm; and, (**g**–**i**) 0.10 µm.

Figure 5a–i depict a series of optical images of a cracked track. For a stretched track with *t*_track_ = 0.04 µm (the calculated ratio of the elongation stiffness was 0.03), several smaller cracks were observed in the track (Figure 5a–c). When the elongation rate was 10%, few cracks were caused in the track, as shown in Figure 5a. As the elongation rate increased, new cracks were caused, and the number of cracks increased (Figure 5b,c). This indicates that a crack configuration with *t*_track_ = 0.04 µm is a multiple-crack, corresponding to the results of Table 1. In this case, the track might be conductive even if the track was stretched up to several tens percent as shown in a previous study [15]. For *t*_track_ = 0.53 µm (0.35 is the ratio of the elongation stiffness), several larger cracks were observed in the track, as shown in Figure 5g–i. The cracks propagated along the track-width direction, which was nearly perpendicular to the stretching direction. The crack width increased with the increase in the elongation rate; whereas, the number of cracks was nearly constant. For *t*_track_ = 1.17 µm (0.78 is the ratio of the elongation stiffness), a similar trend was observed. This indicates that the crack configuration for *t*_track_ = 0.53 µm and 1.17 µm is single-crack growth type, and also corresponds to the results of Table 1. In this case, the track might lose its conductivity, even when the elongation rate was under several percent, because of the larger crack propagation. For a cracked track with *t*_track_ = 0.10 µm and 0.18 µm, an intermediate type of crack configuration was observed, as shown in Figure 5d–f. Many microcracks were caused, and each crack propagated, as the elongation rate increased.

**Figure 6:**
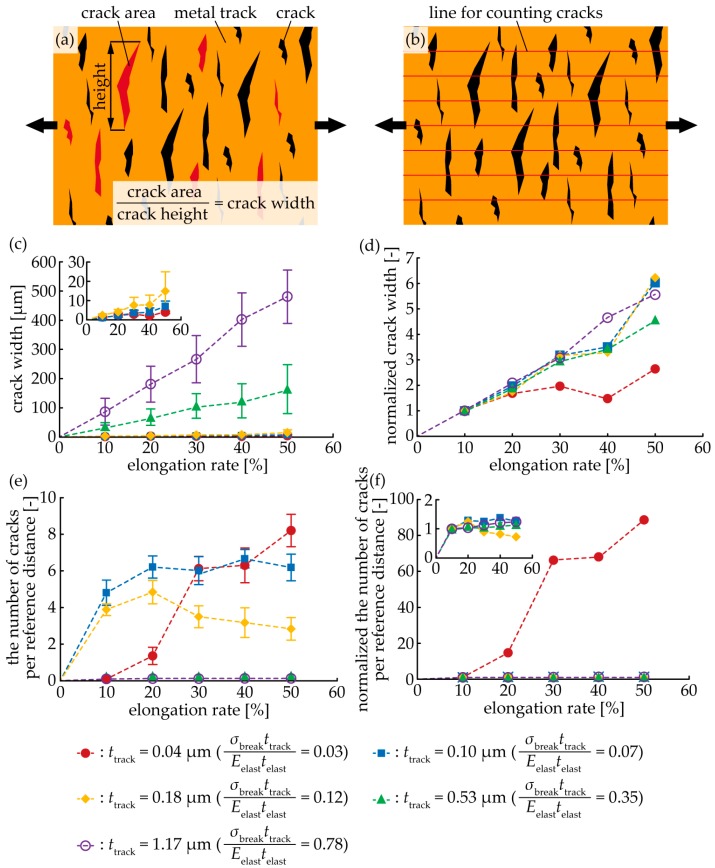
Schematic of the (**a**) measurement of the crack width and (**b**) number of cracks. Relationship between the (**c**) crack width and elongation rate; (**d**) normalized crack width and elongation rate; (**e**) number of cracks and elongation rate; and, (**f**) normalized number of cracks and elongation rate. The reference distance was 100 µm for an elongation of 0%.

For better understanding, numerical analysis on the crack width and number of cracks was conducted. The crack width and number of cracks were measured from the optical image of a cracked track (Figure 6a,b), and the transition of the crack width and the number of cracks were analyzed. The crack width was measured as follows: seven cracks were randomly selected on the optical image of a cracked track, and the values of each crack area were measured. The crack width was obtained by dividing each area by the height of each crack. Hence, the crack width is the average value, along the stretching direction. The number of the cracks was calculated as follows: seven lines was drawn on an optical image of a cracked track at regular intervals, and the number of cracks, across the line, were counted. The direction of the line was along the stretching direction, and the line was drawn end-to-end on the optical image. The counted number was divided by the reference distance, which was 100 µm for an elongation of 0%. Therefore, the number of cracks is the average value of the reference distance. The crack widths for *t*_track_ = 0.04 µm, 0.10 µm, and 0.18 µm ranged from several micro to several tens of micrometers, as shown in Figure 6c. On the other hand, for *t*_track_ = 0.53 µm and 1.17 µm, the crack widths ranged from several tens to hundreds of micrometers. Figure 6d shows the normalized crack width by the value of the crack width at 10% elongation rate. For *t*_track_ = 0.04 µm, the normalized crack width ranged from 1 to 2. On the other hand, for *t*_track_ = 0.10 µm, 0.18 µm, 0.53 µm, and 1.17 µm, the normalized crack width increased almost linearly, as the elongation rate increased. This indicates that in the case of the multiple-crack growth type, the crack width is nearly constant or increases marginally, as the elongation rate increases; whereas, in the case of a single-crack growth type, the crack width increases almost linearly. The number of cracks per reference distance was more than one, for *t*_track_ = 0.04 µm, 0.10 µm, and 0.18 µm (Figure 6e). On the other hand, for *t*_track_ = 0.53 µm and 1.17 µm, the number of cracks were approximately zero. Figure 6f shows the normalized number of cracks by the value of the number of cracks at a 10% elongation rate. For *t*_track_ = 0.04 µm, the normalized number of cracks increased from approximately 1–90, as the elongation rate increased. On the other hand, for *t*_track_ = 0.10 µm, 0.18 µm, 0.53 µm, and 1.17 µm, the normalized number was nearly constant, at unity. That indicates that, in the case of the multiple crack-growth type, the number of cracks increases suddenly, as the elongation rate increases; whereas, in the case of the single-crack growth type, the number is nearly constant.

### 3.2. Wave-Shaped Track

**Figure 7:**
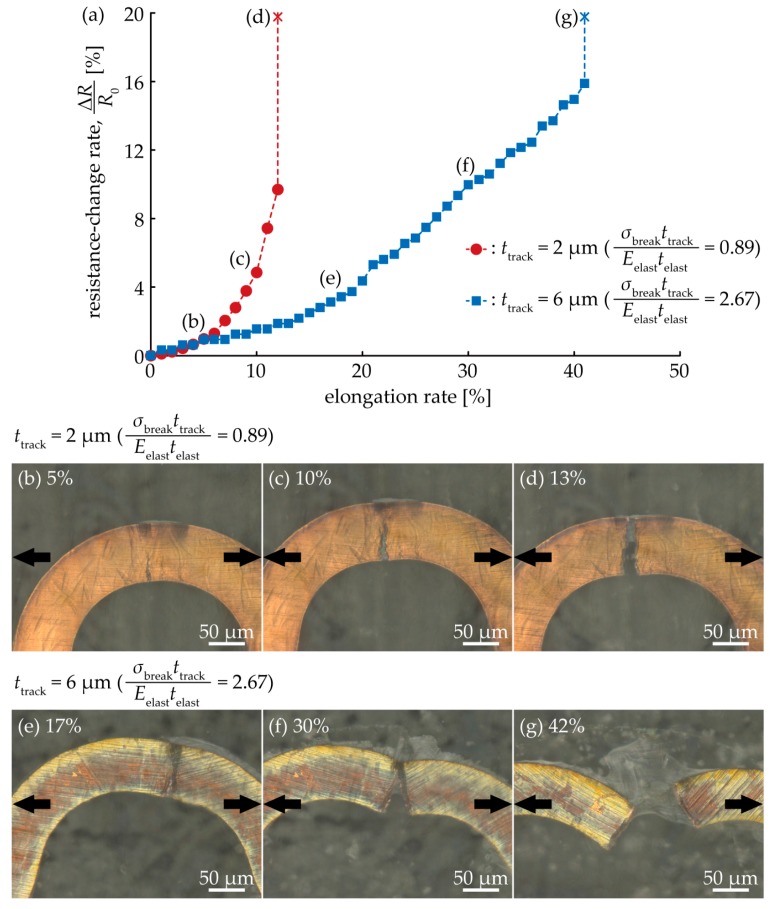
(**a**) Relationship between the resistance-change rate and elongation rate for *t*_track_ = 2 µm and 4 µm; Optical images around the apex of a wave shape for (**b**–**d**) *t*_track_ = 2 µm and (**e**–**g**) *t*_track_ = 6 µm.

Figure 7a shows the resistance-change rate in terms of the elongation rate, for *t*_track_ = 2 µm and 6 µm (0.89 and 1.78 are the calculated ratios of the elongation stiffness, respectively). In both cases, the resistance rate increased, as the elongation rate increased, and each track completely broke at 13% and 42% of the elongation rate for *t*_track_ = 2 µm and 6 µm, respectively. Figure 7b–g show the optical images of the crack propagation of each track. When strain was gradually applied to the track, some crack initiations were observed (Figure 7b,e), around the apex of the wave shape. Each crack gradually propagated as the elongation rate increased (Figure 7c,f), along the direction of the track width. This indicates that the resistance rate was increased by crack propagation. The tracks were completely broken, when the crack propagated completely (Figure 7d,g). The crack configuration was the single-crack growth type, in both cases, corresponding to the results of Table 1. When compared with *t*_track_ = 2 µm and 6 µm, the crack propagation speed was different. Figure 7a–g indicate that the crack propagation speed at *t*_track_ = 2 µm was faster than that at 6 µm. 

**Figure 8:**
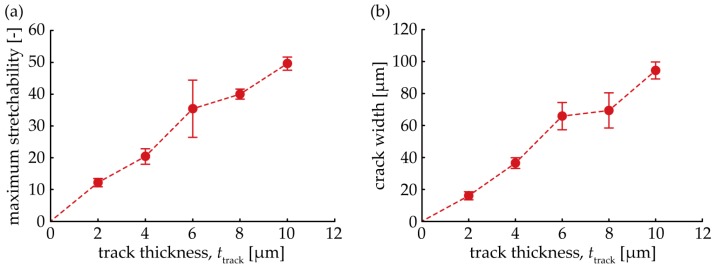
Relationship between the (**a**) maximum stretchability and track thickness; *t*_track_, and the (**b**) crack width and track thickness, *t*_track_.

Figure 8a shows the relationship between the maximum stretchability of a wave-shaped track and the track thickness, *t*_track_. The number of trials for each thickness was five. The values of the maximum stretchability were 12%, 20%, 35%, 40%, and 50% at 2 µm, 4 µm, 6 µm, 8 µm, and 10 µm of *t*_track_, respectively; therefore, the maximum stretchability increased proportionally, as *t*_track_ increased. This indicates that the crack propagation speed was reduced, as *t*_track_ increased; hence, the maximum stretchability increased. In addition to this, we consider that the maximum stretchability may also be increased by decreasing the width or wavelength of the wave-shape as shown in previous studies [19,22]. Figure 8b shows the relationship between the crack width and *t*_track_. The number of trials for each thickness was five, again. The crack was trapezoid-shaped; therefore, the crack width was defined as the distance between the mid points of the sides. The values of the crack width were 16 µm, 36 µm, 66 µm, 69 µm, and 94 µm at 2 µm, 4 µm, 6 µm, 8 µm, and 10 µm of *t*_track_, respectively; hence, the crack width increased proportionally, as the track thickness increased. This indicates that a larger strain energy was caused, as the track elongation rate increased, and this energy was released when the track was broken. This larger energy renders the crack wider.

## 4. Conclusions

We analyzed the factors determining the crack configurations of a stretched metal conductive track embedded in stretchable elastomer, by both modeling and experiments. The modeling analysis indicated that the crack configuration is determined by the ratio of the elongation stiffness of the track and the elastomer, and it is classified into two types: multiple-crack growth and single-crack growth. We established that the multiple-crack growth type is often observed, when the ratio of the elongation stiffness is lesser than approximately 0.1, and a single-crack growth type is often observed, when ratio is more than approximately unity, with reference to previous studies. In the experiments, to verify the modeling analysis, two conventional metal-track shapes were examined: straight-shaped tracks with *t*_track_ = 0.04–1.17 µm (0.03–0.78 is the ratio of the elongation stiffness), and wave-shaped tracks with *t*_track_ = 2–10 µm (0.89–4.45 is the ratio of the elongation stiffness). For straight-shaped tracks, the multiple-crack growth type was observed, when *t*_track_ = 0.04 µm, and the crack configuration gradually changed to a single crack, as *t*_track_ increased. This corresponds to the result of the modeling analysis. For wave-shaped tracks, only a single-crack growth type was observed; hence, this also corresponds to the modeling analysis. In addition, for a wave-shaped track, the crack propagation speed was reduced and the maximum stretchability of the track increased linearly, with the increase in *t*_track_. 

## Figures and Tables

**Table 1:** Relationship between the calculated values of the ratio of the elongation stiffness and the crack configurations, in previous studies and this study.

Ref.	Structure	$\frac{\sigma_{\mathrm{break}}t_{\mathrm{track}}}{E_{\mathrm{elast}}t_{\mathrm{elast}}}$	Crack Configuration
[13] (straight-shaped track)	*t*_track_ = 0.05 µm (gold) *t*_elast_ = 1 mm (PDMS)	0.01	Multiple-crack growth type
[16] (straight-shaped track)	*t*_track_ = 0.05–0.1 µm (gold) *t*_elast_ = 1 mm (PDMS)	0.01–0.02	
[14] (straight-shaped track)	*t*_track_ = 0.075 µm (gold) *t*_elast_ = 0.3 mm (PDMS)	0.06	
[12] (straight-shaped track)	*t*_track_ = 0.035 µm (gold) *t*_elast_ = 0.12 mm (PDMS)	0.07	
[15] (straight-shaped track)	*t*_track_ = 0.04 µm (gold) *t*_elast_ = 0.076 mm (PDMS)	0.12	
[19] (straight-shaped/wave-shaped track)	*t*_track_ = 2.5–5 µm (gold) *t*_elast_ = 0.4 mm (PDMS)	1.44–2.88	Single-crack growth type
[21] (wave-shaped track)	*t*_track_ = 18 µm (copper) *t*_elast_ = 1 mm (PDMS)	2.76	
[20] (wave-shaped track)	*t*_track_ = 17 µm (copper) *t*_elast_ = 0.1 mm (PDMS)	26.15	
This study (straight-shaped track)	*t*_track_ = 0.04–1.17 µm (copper) *t*_elast_ = 0.1 mm (PU)	0.03–0.78	Multiple-crack growth/Single-crack growth type
This study (wave-shaped track)	*t*_track_ = 2–10 µm (copper) *t*_elast_ = 0.1 mm (PU)	0.89–4.45	Single-crack growth type
